# Record-keeping: Challenges experienced by nurses in selected public hospitals

**DOI:** 10.4102/curationis.v41i1.1931

**Published:** 2018-07-30

**Authors:** Takalani E. Mutshatshi, Tebogo M. Mothiba, Pamela M. Mamogobo, Masenyani O. Mbombi

**Affiliations:** 1 Department of Nursing Science, University of Limpopo, South Africa

## Abstract

**Background:**

Patients’ records provide a trace of care processes that have occurred and are further used as communication amongst nurses for continued management of patients. Nurses have the responsibility to ensure that records are accurate and complete in order to effectively manage their patients. In hospitals, nurses have to record a wide range of information in the patient’s records and this leads to increased workload on the part of nurses that compromises accurate record-keeping.

**Objectives:**

The purpose of this study was to explore and describe the challenges experienced by nurses with regard to record-keeping at selected public hospitals in the Vhembe district, Limpopo Province, South Africa.

**Method:**

A qualitative, explorative and descriptive research design was used. Nurses working in selected public hospitals were purposively selected and semi-structured interviews were conducted until data saturation was reached. Data were analysed using the Tesch’s open-coding method.

**Results:**

Nurses working in public hospitals experience record-keeping as a challenging activity owing to a variety of challenges which include lack of time to complete the records, increased patients’ admission and shortage of recording material.

**Conclusion:**

Record-keeping is not done properly which is problematic, and it is recommended that there should be continuous training, monitoring and evaluation of nurses on record-keeping issues, supply of adequate recording materials and proper time management amongst nurses to improve record-keeping challenges. The need for comprehensive record-keeping remains fundamental in public hospitals in order to improve patient care.

## Introduction and background

Good nursing practice requires detailed record-keeping that is comprehensive, timely and accurate. Without complete recording there is no evidence to prove that care was provided to the patient, and in nursing practice there is a saying that ‘what is not recorded has not been done’ (Marinic 2015; Taiye 2015). Furthermore, poor record-keeping not only undermines patient care but makes the nurses more vulnerable to legal claims which arise from breakdown in communication that results from incomplete or inadequate records (Marinic 2015). The South African Nursing Council (SANC) Rules and Regulation R387 relating to Acts and Omissions requires a nurse to keep clear and accurate records of all nursing actions done to the patient at all times and failure to do so constitutes a professional misconduct where the SANC may take disciplinary action against such nurses (SANC 2005, R387 as amended).

Mathloudakis et al. (2016) found that sub-standard documentation of nursing actions is associated with prolonged hospital stay of the patients and increased patient mortality. Thus, poor record-keeping practices amongst nurses lead to breakdown in communication amongst health care professionals. Poor record-keeping does not put the patient at the centre of care but increases medico-legal risks and hinders tracking of clinical care decisions and care goals. Despite numerous efforts by nurse managers to improve record-keeping, inadequate recording remains a global challenge in public hospitals which is frequently reported in research findings of many nurse researchers (Okaisu et al. 2014).

A study conducted by Genctuc et al. (2017) revealed that nurses do not record their actions to a great extent and they only record observations when there are abnormalities and such incomplete recording may lead people to think that they did not fulfil their duties. Okaisu et al. (2014) conclude that recording consumes up to 50% of nurses’ time per shift even though it serves a number of important functions, such as communication amongst health care workers for continuity of care, and is essential for the provision of safe and effective care and protection of accountability of nurses who delivered that care. The study further recommends that in order to achieve improved recording in nursing practice, continuous leadership support to nurses in hospitals is important.

The SANC’s analysis report from 2003 to 2008 revealed that 769 nurses were found guilty of professional misconduct, with 587 professional nurses being charged amongst others with failure to record their nursing actions in the patient record (Van Graan, Williams & Koen 2016). News24 (2013) from the South Africa Broadcasting Corporation reported on an incident where a lawsuit required the Limpopo Health Department to pay about R1.7 million for negligence as a consequence of the nurses failing to make a complete recording of nursing interventions for a patient admitted in a certain hospital. A study conducted in Vhembe district revealed that patient records were incomplete and some information was never recorded, despite availability of recording forms (Shihundla, Lebese & Maputle 2016).

## Problem statement

Nurses providing care in all health care institutions including hospitals are expected to record all nursing actions in numerous recording forms such as the nursing process forms. A study conducted by Khani et al. (2016) revealed that fatigue, large number of patients, high volume of nursing actions, lack of continuous monitoring and evaluation, lack of reward system to staff by nursing management were important factors affecting nursing records in hospitals. Ahn, Choi and Kim (2016) also identified that work experience of nurses and nature of nursing shifts are other factors that influence timeous record-keeping in public hospitals. Currently in public hospitals of Vhembe district in Limpopo Province, the nursing audit of patient records for quality assurance purposes, peer review team meetings, mortality reviews and hospital management meetings continuously led to complaints about the trend of poor record-keeping, despite all efforts to improve record-keeping challenges. It was against this background that the study aimed to explore the challenges experienced by nurses with regard to record-keeping at selected hospitals.

## Purpose of the study

The purpose of the study was to explore and describe the challenges experienced by nurses with regard to record-keeping at selected public hospitals.

## Definition of key concepts

A *nurse* means a person registered in a category under section 31(1) in order to practise nursing or midwifery (Nursing Act 2005). In this context, a nurse is a professional nurse who is trained to render nursing care and keep records of nursing care in a public hospital.

A *challenge* is regarded as a very difficult situation with a variety of conditions or circumstances at a given time (Matlakala, Bezuidenhout & Botha 2014). In this study, challenges are those problems experienced by nurses with regard to record-keeping when caring for patients at public hospitals.

*Experiences* are those occasions and details that a person has observed and come across in life that create a long-lasting impression (Soanes & Stevenson 2008). In this study, experience is the feelings, observations and approaches of nurses with regard to record-keeping.

*Record-keeping* is the process of making an entry or storing of information on patient records in order to promote and maintain patient safety (Taiye 2015). In this study, this is the adequate and complete recording of all activities that the nurse has done on the patient.

## Contribution to the field

The information obtained from challenges experienced by nurses in record-keeping can help hospital management to support nurses in practice to improve recording.

## Research method and design

A qualitative approach with explorative and descriptive research designs was followed. The design was appropriate for this study as it provided nurses with the opportunity to explain and describe their experiences with regard to record-keeping.

### Context of the study

The study was conducted at selected public hospitals in Vhembe district, Limpopo Province, South Africa.

### Population and sampling

The study was conducted in selected public hospitals in the Vhembe district, Limpopo Province of South Africa. The total population of nurses in the selected hospitals was 183. Non-probability purposive sampling was used to select 13 participants. The participants included professional nurses from the medical, surgical and paediatric wards in the selected public hospitals.

### Data collection

Data were collected through semi-structured interviews using an interview guide. The interviews were conducted over a period of 2 months. The questions were formulated in English as participants were only professional nurses. These questions assisted the researchers to probe more into the challenges experienced by nurses with regard to record-keeping to elicit more information, which was important for the study. Participants were included in the interviews based on their willingness to participate in the study. Field notes were written to capture the non-verbal cues and a voice recorder was used with the permission of participants to record all interview sessions conducted. Member checking was done to audit the information collected from the participants to confirm whether the notes were a true reflection of the challenges they experienced with record-keeping.

### Data analysis

Data were analysed using the Tesch’s descriptive analysis method for qualitative research as outlined by Creswell (2014). The audio tapes of the interviews were transcribed verbatim. The researchers read through transcriptions and wrote ideas to get a sense of the information as a whole. A list of all topics and similar topics were clustered together and were arranged in major and unique topics. The list was compared with the original data. Abbreviations of topics as codes were made and codes were written next to the appropriate segment of the text. Transcripts were coded using an independent coder and themes were identified.

### Trustworthiness

The four criteria to ensure trustworthiness as outlined by De Vos et al. (2011) were used to establish the truth value of the study. Credibility was ensured through triangulation in data collection where interviews were conducted, data audio were recorded and field notes were taken. Audio tapes and field notes were kept as part of the audit trail. The researcher also did member checking to clarify errors and confirm findings where participants were given a chance to confirm their responses during the interview process. In this study, transferability was not possible as the study was conducted only in Vhembe district of Limpopo Province.

Dependability was ensured by outlining the detailed methodology, data collection using the voice recorder and taking of field notes. The methodology, results and recommendations were also discussed with peers who are experts in qualitative research and who were not involved in the study. Confirmability was achieved through the use of an independent coder to confirm the themes and field notes and audio recordings were checked with experts in qualitative research. In this study, ‘bracketing’ was used by the researchers to ensure dependability. The researcher identified and set aside preconceived ideas and beliefs about record-keeping in order to minimise bias.

### Ethical considerations

Ethical clearance was obtained (MREC/HS/265/2014; PG) before the study was conducted. Permission to conduct the study was obtained from the Provincial Department of Health and Chief Executive Officers of participating hospitals. Participants were briefed and given information prior to the data collection process to allow them to make an informed voluntary decision whether to participate in the study. Informed written consent was obtained from the participants after all possible information on the goal of the investigation and procedures were explained. Confidentiality was ensured in that no names were reflected and audiotape recordings were transferred to a personal laptop which is password protected and put under lock and key in the researcher’s office. Right to self-determination was ensured in that participants were not forced to participate in the study. The researcher explained the purpose and use of audio recording and note taking during the interview sessions. Data protection was achieved by ensuring that results, audio-recorded data, transcribed verbatim, and field notes were kept safely in a locked cabinet in the researcher’s office to prevent access to information by any other person.

## Presentation of results

The following three themes were identified as challenges experienced by nurses with regard to record-keeping in selected public hospitals:

- time needed to complete recording forms
- patient movement related to increased patient admission
- inadequate supply of recording materials leading to incomplete recording.

## Discussion of findings

The discussion of the findings is based on the data collected through semi-structured interviews conducted with 13 participants on the challenges they experienced with record-keeping. Although recording is integral in nursing practice, nurses indicated that recording of all activities was difficult owing to the following challenges: lack of time to complete the records, increased patient movement such as admission of patients or work overload and inadequate supply of recording material leading to incomplete recording. Three themes that emerged are described below in narrative form as challenges experienced by nurses with regard to record-keeping.

## Challenges related to lack of time to complete the records

Nurses experienced record-keeping challenges in patient care related to the many nursing actions and interventions that they carry out on the patients. There are many forms that the nurses have to complete and keeping such records is laborious and time consuming. Nurses have to record everything that they do to a patient and it is time consuming; thus, professional nurses experienced time factor as a challenge to good record-keeping. There are many forms that are to be written manually and this poses a challenge to nurses as they end up not completing all forms, resulting in incomplete recording.

This was confirmed by Participant 1 who indicated that:

‘Hmmm, like when admitting, it takes about an hour to complete all the forms for only one admission, there is too much writing and we cannot complete all those many forms.’ (Participant 1, female, professional nurse)

Participant 2 also said that:

‘There may be three patients for admission and you are giving injections and taking vital signs. It is not possible to utilise [*the*] record everything you have done due to lack of time, it is too long and time consuming. No, there is some negative experience; it’s time consuming because it has many things to consider. It needs time to complete the forms. For one patient, you can spend 30 minutes to an hour on one patient.’ (Participant 2, female, professional nurse)

One of the limitations to record-keeping is the time factor, as there is no adequate time to do the recording after all activities are done in a clinical setting. Mahony et al. (2014) agree with the findings of this study in that excessive time is needed for recording which leaves reduced time for patient care. Furthermore, nurses mention lack of sufficient time for recording all the care implementation as one of the most important barriers. Okaisu et al. (2014) in Uganda alluded that the forms to be completed during recording required much writing and relied on nurses’ recall of what to document, leading to incomplete recording.

Kamau (2015) is of the opinion that to overcome the challenge of lots of writing, nurses’ performance can be improved by using well-designed computer technology to document care, which can improve the speed and quality of documentation, resulting in more time for direct patient care. The study further indicates that the challenge with such swift recording may only be managing the change from paper to electronic records in a constructive and supportive way to alleviate resistance amongst nurses (Kamau 2015).

## The challenge related to increased patient movement leads to lack of recording

Nurses continually cite increased number of admitted patients as an important reason as to why the recording is not implemented effectively at hospitals. Staff shortages impact negatively on record-keeping as few nurses attending many patients have to record in many forms. During interviews, professional nurses indicated that they are overworked, as they have to do lot of work related to patient care and this leads to poor recording of such activities in the patient’s file. Participants also reflect that the increased workload is associated with shortage of staff and admission of large numbers of patients. This observation was confirmed by a participant who told that:

‘Yes, we are not having enough staff here. Complete recording is difficult especially when we are admitting many patients, it will be difficult to record due to shortage of staff.’ (Participant 3, male, professional nurse)

Another participant added:

‘So, in our institution, you find that we are very much short staffed and you are required to fill all those different forms and it is difficult when time is limited, so there is a lot of workload.’ (Participant 4, female, professional nurse)

Another participant stated that:

‘there is too much workload and we are short-staffed, nurses are to bath, feed patients, do bed making, give medications and attend to emergencies, this is too much, the recording is another problem, this is why records are not fully completed.’ (Participant 1, female, professional nurse)

There are many factors ranging from personnel shortage and negative attitude of nursing personnel towards recording; nurses perceive that they spend much time on manual recording, leading to incomplete recording and so the requirement for more staff (Mahony et al. 2014). The findings of this study are congruent with the findings of a study conducted by Jooste, Van der Vyfer and Van Dyk (2010) which indicate that nurses continue to report the shortage of staff as a contributing factor to non-implementation of the nursing process including incomplete recording.

Aseratie, Murugan and Molla (2014) support the findings of this study that nurses are not recording because of work overload and that nurses encounter major barriers to documentation owing to mismatches between staffing resources and workload. The study further indicates that when nurses experience extra workload, this predisposes them to decreased morale and inadequate work practices, including poor recording practices, which puts pressure on the quality of care rendered to patients. Shihundla et al. (2016) agree with the study findings outlining that nurses find it difficult to cope with the increased workload associated with documenting patient information on the multiple records that are utilised at heath facilities, leading to incomplete information documented on patient records. These authors recommend that the number of nurses at facilities should be increased to reduce the increased workload.

Wang et al. (2016) indicate that in most low- and middle-income countries, lack of recording and systems is still a major obstacle in measuring the quality of health care. Nurses are increasingly being made aware of the role of clinical records in health care litigation despite the shortage that they are experiencing, but nurses must ensure that their notes are ‘meticulous’ from a legal perspective because an activity that is not documented is considered as not done. Inan and Dinc (2013) also confirm that keeping good records is regarded as an essential professional and legal requirement of being a nurse and postponement of documentation of patient information immediately after the event has occurred might lead to medico-legal hazards.

## The challenge related to inadequate supply of recording materials, leading to incomplete recording

Nurses experience challenges with record-keeping in public hospitals owing to inadequate provision of recording material. This study found that nurses were able to perform various activities and plan for patient care, but such activities are not completely recorded owing to a lack of recording materials. There are inadequate recording forms available.

Another participant indicated that:

‘Hmmm, even though sometimes when I say time covers a lot of things that we experience because sometimes when you want to record you have to run around, there are is [*sic*] stationery to record on. Therefore, we need to move from one ward to the other while patients are waiting for you to come back and you won’t because you still looking for materials that you will write on.’ (Participant 5, female, professional nurse)

Another participant told that:

‘Yes we are having problems like shortage of stationery which we are coming across. Sometimes you find is difficult to record everything on different forms because of shortage of stationery. Sometimes we collect our own money to buy papers for photocopying because there is no stationery in the wards. Other things we end not completing the recording and the audit people blames us.’ (Participant 4, female, professional nurse)

The response implies that nurses experience challenges in recording the planned interventions in order to achieve the set goals. Gilson (2014) also agrees with this study finding that unavailability of recording documents also poses a challenge to nurses making recording difficult. Nurses perceive the shortage of supplies and dysfunction or absence of equipment as a key obstacle to quality patient care. The findings of this study are supported by Mahmoud and Bayoumy (2014) who also indicate that many nurses complain of insufficient resources, thus unavailability of equipment and supplies including recording material, searching for supplies and having to wait for other recording material are performance barriers during provision of care to patients. Afolayan et al. (2013) reveal that unavailability of materials for documentation is a challenge that affects record-keeping in hospitals. Nurses need charts for effective practice of nursing care at a hospital, and these charts are unavailable and hence record-keeping is affected.

## Limitations of the study

The challenges experienced by nurses in record-keeping were from nurses working in selected public hospitals in the Vhembe district of Limpopo Province; therefore, the study findings have limited application to the hospitals and professional nurses who were included in this study.

## Recommendations

The study recommends continuous nursing management support to nurses working in the hospitals through provision of adequate recording materials to promote effective recording. The study further recommends continuous training, monitoring and evaluation of nurses on record-keeping and its importance, and this can improve record-keeping practice in hospitals. The study also recommends that hospital management arrange time management workshops for nurses so that they can effectively manage their time when caring for their patients.

## Conclusion

It was evident from the study findings that nurses have challenges during the recording of nursing actions provided to the patient owing to various factors. The results further pointed out that recording of planned nursing interventions are not completely carried out owing to too much time needed for recording, increased patients’ admission and inadequate supply of recording material; therefore, nurses need to be motivated on the importance of recording when implementing nursing actions in patient care.
